# Antibiotic Irrigation: A Promising Unconventional Method for Facial Carbuncle

**DOI:** 10.7759/cureus.14710

**Published:** 2021-04-27

**Authors:** Jia Ji Ng, Hardip Gendeh, Hui Yan Ong, Shashi Gopalan, Juani Hayyan Abdul Karaf

**Affiliations:** 1 Otolaryngology - Head and Neck Surgery, University Kebangsaan Malaysia Medical Center, Kuala Lumpur, MYS; 2 Otolaryngology - Head and Neck Surgery, University Malaysia Medical Centre, Kuala Lumpur, MYS; 3 Otolaryngology - Head and Neck Surgery, Tengku Ampuan Rahimah Hospital, Klang, MYS

**Keywords:** facial carbuncle, conservative treatment, antibiotic irrigation

## Abstract

Carbuncle is conventionally treated with combinations of intravenous antibiotics and surgical intervention; be it saucerization or incision and drainage. Cosmesis outcome might be unfavorable following surgical intervention, especially when the facial region is involved. Skin grafting surgery may even be needed as a second-stage procedure for a larger wound. We reported a series of three facial carbuncles treated successfully with a new improvised method. Our method includes performing a stab incision prior to draining of pus, coupled with minimal wound debridement, followed by regular irrigation of the wound with antibiotics containing solution. Based on the three cases reported in this article, we conclude that this method is more superior as there is more skin preservation, better patient tolerance, shorter hospital stays, and favorable cosmesis outcome.

## Introduction

Carbuncle is conventionally treated with combinations of antibiotics and surgical intervention; be it saucerization or incision and drainage (I&D), and sometimes followed by split skin graft for closure of the wound [1,2]. They are usually caused by Staphylococcus aureus-infected furuncles. Cosmesis outcome might be unfavorable following surgical intervention, especially if it is a large wound or facial region is involved. Ngui et al. suggested a more conservative approach by implementing minimal wound debridement followed by thrice daily irrigation of antibiotic-containing solution into the wound, with the aim of maximizing skin conservation and thus a better cosmesis outcome [3]. We modified this technique and reported our local experiences with a series of cases of facial carbuncle being treated successfully with our method.

## Case presentation

Case 1

A 52-year-old gentleman with underlying diabetes mellitus, hypertension, and end-stage renal failure requiring regular hemodialysis was admitted for right facial swelling of one-week duration. It initially started as a pimple-like lesion for two days, and progressively worsened to affect his entire right side of the face. 

**Figure 1:**
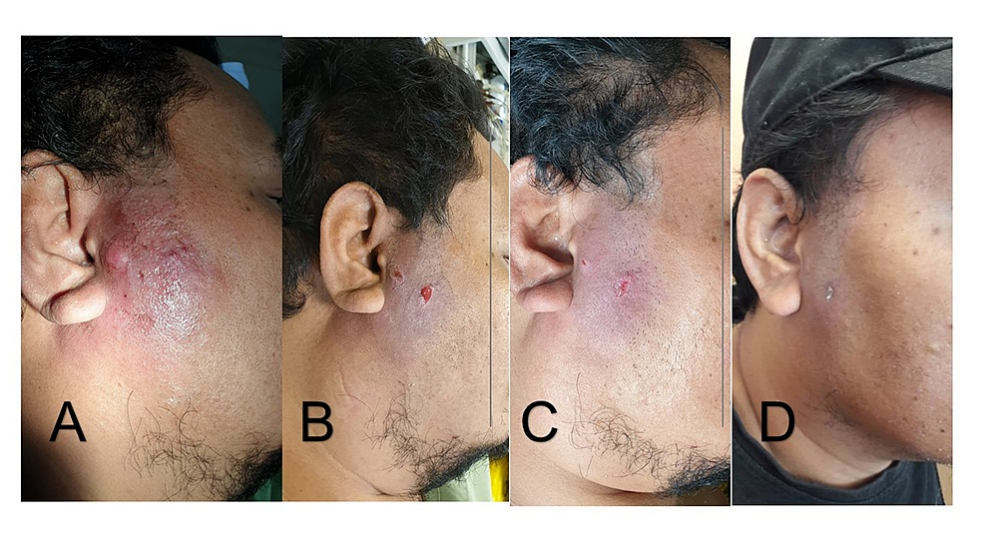
Right facial carbuncle of Case 1 (A) Right facial carbuncle during admission. (B) Day 1 post wound irrigation – induration, erythema and swelling reducing. (C) Day 3 post wound irrigation – surrounding induration much reduced. (D) Well healed wound three weeks after admission.

Upon clinical examination, he was febrile at 38.5 degrees Celsius and his random blood sugar level was 18mmol/L. There was a large carbuncle over the right side of the face measuring 10x8cm and it extended posteriorly to the angle of the mandible and superiorly to the lateral cantus of the right eye (Figures 1A-1D). It was tender, tense, erythematous with multiple sinus pus discharges.

In view of the patient’s septic condition and underlying multiple comorbidities, which rendered him to be of high risk for general anesthesia (GA), he was admitted to the hospital and commenced on intravenous amoxicillin with clavulanic acid 1.2gm and metronidazole 500mg three times a day. His blood sugar level was closely monitored and optimized. Strict diabetic diet and education were also given to the patient to aid his recovery.

Instead of performing I&D under GA, a small stab incision was made over the carbuncle side under local anesthesia. The incision drained some pus and at the same time, served as a portal for wound irrigation. The irrigation solution used was gentamycin 80mg diluted in 500mL of normal saline. A 20-cc syringe was used to irrigate the wound through the incision site. The wound was irrigated twice a day, 12 hours apart, for the first three days. We observed improvement of the wound following the irrigation regimen. With the surrounding induration reduced, and there was no more pus observed; once-daily irrigation was performed for two more days. He completed intravenous antibiotics for a total of five days and was discharged home with oral antibiotics to complete a total of two weeks' duration.

Culture from the wound grew Staphylococcus aureus, which was sensitive to the antibiotics given. His wound healed with minimal scarring by the third week.

Case 2

A 64-year-old lady with underlying diabetes mellitus, hypertension, and dyslipidemia presented with right cheek swelling for the past four days. Her right cheek swelling was increasing in size, painful with pus discharge. There was no history of fever, facial asymmetry or having difficulty in mouth opening. She also denied a history of trauma or insect bite.

**Figure 2:**
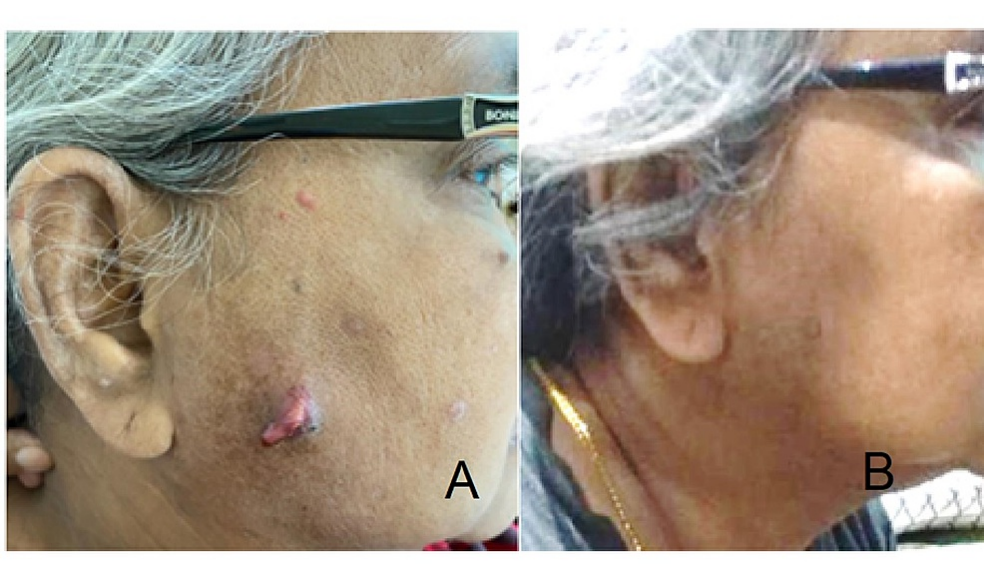
Right facial carbuncle of Case 2 (A) Right facial carbuncle during admission – surrounding erythema and pus discharging from the punctum. (B) Well-healed wound three weeks after admission.

On examination, there was a 5x4cm right cheek swelling which was tender, erythematous, and with multiple discharging punctum (Figures 2A, 2B). Facial nerve and otoscopy examination were normal. Her random blood glucose was 19mmol/L. Blood investigations predictably revealed high septic parameters; with total white cell counts of 15.4x109/L and C-reactive protein of 80.13mg/L.

She was admitted to the ward for intravenous antibiotics as well as glycemic control. The multiple punctums were actively discharging, thus no stab incision was made. The wound was debrided and irrigated with saline mixed with gentamycin solution. She was initially started on intravenous ampicillin/sulbactam 1.5g; however, she developed an allergic reaction and was subsequently converted to intravenous Clindamycin 300mg four times daily. As the debrided wound was clean and the induration was minimal, irrigation was only performed once daily for this patient and she tolerated the procedure well. Irrigation was done for the next three days and the induration and wound improved. She was discharged well after day 4 of admission, with oral antibiotics.

Culture and sensitivity of the pus from the cheek swelling grew Staphylococcus aureus, which was sensitive to the antibiotics prescribed. She completed a total of two weeks of antibiotics and during her outpatient clinic follow-up, the wound recovered well with minimal scarring.

Case 3

A 46-year-old gentleman with underlying hypertension and diabetes mellitus on oral hypoglycaemic agent presented to ENT clinic with the complaint of painful left preauricular swelling for one week. The swelling initially started off as a blister and it gradually worsened with pus discharging over the week. This was his first episode of similar swelling and he denied a history of trauma nor insect bite prior. There was no fever, no ear discharges, no hearing loss, and no other systemic symptoms.

**Figure 3:**
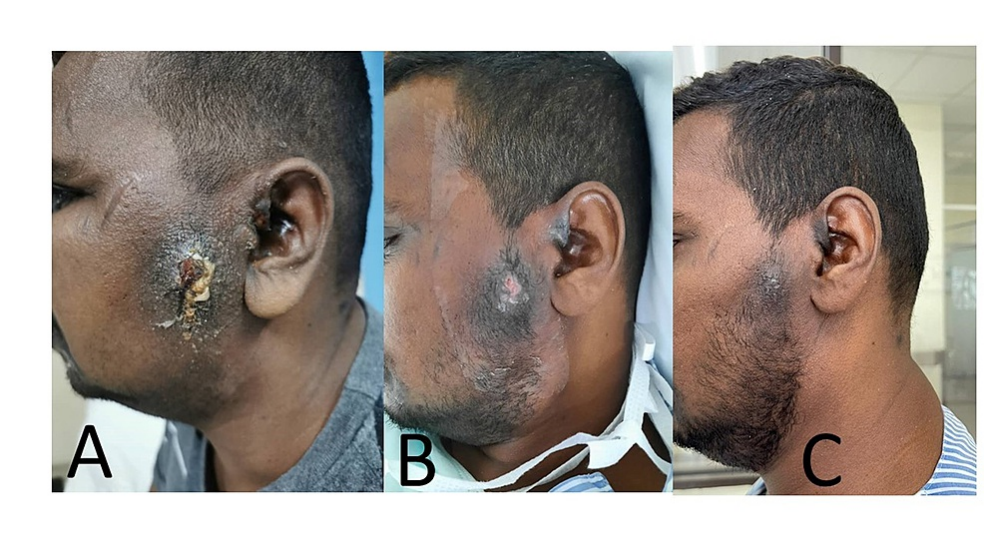
Left facial carbuncle of Case 3 (A) Left facial carbuncle with pus discharging and indurated surrounding skin during admission. (B) Left facial carbuncle after two days of irrigation. (C) Resolving left carbuncle with minimal scarring on the second week.

On examination, he was comfortable and non-septic looking. There was a left preauricular swelling of 5x5cm seen, which was firm, with multiple punctums and pus discharge (Figures 3A-3C). It was warm and tender on gentle palpation. There was left cervical lymph nodes of 1x2cm. Otoscopy examination was normal. There was no significant finding from other systemic examinations.

His random blood sugar was 21.4mmol/L and his white blood cells count was 13.38x109/L. He was admitted to the ward for intravenous amoxicillin and clavulanate 1.2g three times daily. The wound was cleaned, and a stab incision made. Gentamycin irrigation was performed three times a day and he tolerated the procedure well. His symptoms and wound improved after two days and the irrigation was reduced to once daily. During his stay, his sugar was well controlled with continuous intravenous insulin infusion and was subsequently converted to basal-bolus regimen. He completed five days of intravenous antibiotics and with his general condition improving and wound resolving, he was discharged from the ward with oral antibiotics, to complete a total of 14 days of antibiotics.

His pus culture similarly grew Staphylococcus aureus and was sensitive to the antibiotics prescribed. His wound healed well with minimal scarring on the second week of follow-up.

## Discussion

Furuncle is defined as an infection of the hair follicle with adjacent subcutaneous tissue involvement leading to abscess formation. Carbuncle, on the other hand, is a necrotizing infection of clusters of furuncles that converge into one and drained through multiple follicular openings [4]. Carbuncles are usually seen in patients who are immunocompromised and are recognized as one of the complications of diabetes mellitus. Carbuncles are commonly found on the neck and back; and the majority of patients present late for treatment, up till two weeks after the onset of illness [2,5]. More often, due to patients’ underlying comorbidities, carbuncles could be fatal, especially if treatment is delayed. Sedik reported a huge neck carbuncle with intracranial complications and concluded the need for an aggressive surgical approach for the debridement of necrotic tissues [6].

The most common pathogen in carbuncles is Staphylococcus aureus and can be of methicillin-sensitive or resistance type [1,4]. The pathogen will disrupt the integrity of the skin and subcutaneous tissue; thus, forming communicating abscess with porous openings. The typical clinical characteristics of carbuncles are superficial skin swelling with multiple porous discharges; bounded by patchy necroses which are usually the result of surrounding capillary occlusions [5]. All the cases in our series grew Staphylococcus aureus and was sensitive to the antibiotics given. It was suggested that first-generation cephalosporins, amoxicillin/clavulanate, or Clindamycin should be the first-line antibiotics while waiting for culture results [1].

Treatment for carbuncles requires early administration of antibiotics and timely surgical intervention, as well as optimization of patients’ underlying pre-existing co-morbidities. Reconstructive surgery might be needed later if there is a soft tissue defect following treatment. Two methods of surgical intervention are commonly used, which are saucerization and I&D [7]. Saucerization involves wide excision and removal of the necrotic center and surrounding unhealthy tissue until the surgical margins are viable. The wound is usually large and left to heal with secondary intention or reconstruction surgery if needed. In comparison, I&D has a smaller wound, by only removing the purulent materials in the center and the surrounding inflamed tissues are treated with antibiotics [7]. Saucerization is more preferred as it removes all necrotic tissues in one setting. However, most of the time saucerization is done under GA; and patients with multiple underlying co-morbidities will have to bear high risks for GA. These two methods would require daily dressing and meticulous debridement of unhealthy tissues, which often cause patients a great deal of torment. Following that, some patients might also need secondary procedures sure as secondary suturing or split-thickness grafting for large or non-healing wounds [2].

Ngui et al. proposed a new method of management for facial carbuncle and reported good outcomes from a series of cases [3]. The carbuncle was initially punctured with a needle, necrotic tissue debrided, and then irrigated three times daily with gentamycin solution. The average time for wound healing in the case series is two to four weeks and the average stay in hospital was nine days. For our patients, rather than using a needle, we improvised by making a small stab incision on the necrotic center. This creates an opening and a portal to aid the drainage of pus and necrotic materials and at the same time, for wound irrigation with gentamycin solution. The irrigation solution used was gentamycin 80mg diluted in 500mL of normal saline. The effectiveness of this method was shown in our case studies whereby the swelling, induration, and erythema reduced after two days of regular irrigation and the average stay in hospital was only five days. Comparing to the conventional treatment of surgical treatment followed by skin grating the average days of stay is 21 days, which showed the effectiveness of this method [2].

Irrigation of wounds is an essential step in wound care. Saline is almost always the choice of irrigation be it for clean or dirty wounds. Irrigation with antibiotic-containing solution has also been used for the prevention and treatment of wound infections. In animal studies, these solutions were shown to reduce the rate of surgical wound infections significantly [8]. In a systemic review by Falagas, antibiotic-containing solutions irrigations are used in sutured lacerations; clean, clean-contaminated, and contaminated surgical procedures; as well as intraperitoneal lavage for the prevention of infection [9]. These are also reported to be used as a choice of treatment in mediastinitis, thoracic empyema, peritonitis, prosthetics joint infection, and urinary bladder infections. However, Falagas also recommend more well-designed and randomized studies in the future to gauge the effectiveness and possible toxicity of antibiotic irrigation [9].

In patients with fewer comorbidities, irrigation may be performed in an outpatient setting with oral antibiotics especially with a shortage of beds in the hospital during this pandemic. Healthcare personnel workload may increase as the patient will need to undergo daily wound irrigation up till thrice daily and each cycle may take up to half an hour. However, this process is well tolerated by patients. Nonetheless, it maximizes skin preservation and lesser skin necrosis; thus offering a favorable cosmesis outcome, making it a better treatment option for facial carbuncles.

## Conclusions

The unconventional method of a small central skin incision, antibiotic irrigation with systemic antibiotics is more superior compared to the conventional treatment. Its advantage is shown in its better skin preservation, patient acceptance, favourable cosmesis and most importantly shorter hospital stay.
